# Diverse CBX family members as potential prognostic biomarkers in non‐small‐cell lung cancer

**DOI:** 10.1002/2211-5463.12971

**Published:** 2020-09-21

**Authors:** Xiaobin Xie, Yue Ning, Jie Long, Hongyan Wang, Xiaowei Chen

**Affiliations:** ^1^ School of Basic Medical Sciences Guangzhou Medical University Guangzhou China; ^2^ Department of Pathology The Second Xiangya Hospital Central South University Changsha China; ^3^ Department of Hematology Guangzhou First People's Hospital South China University of Technology School of Medicine Guangzhou China

**Keywords:** bioinformatics analysis, biomarker, Chromobox 3, Chromobox family members, non‐small‐cell lung cancer

## Abstract

Chromobox (CBX) family members are vital epigenetic regulators that repress the transcription of target genes through chromatin modification. Several studies have investigated the role of CBX family members in cancer. However, the function and prognostic value of diverse CBX family members in non‐small‐cell lung cancer remain largely unknown. In this study, we reveal that CBX family members are overexpressed in non‐small‐cell lung cancer tissue compared with normal lung tissue, with the exception of CBX6. Kaplan–Meier analysis demonstrated that high expressions of CBX1 and CBX3 are correlated with overall survival, disease‐specific survival, disease‐free interval, and progression‐free interval for patients with lung adenocarcinoma (LUAD). Furthermore, regression model analysis suggests that CBX3 may be suitable as an independent prediction factor for overall survival and progression‐free interval in patients with LUAD. In addition, CBX3 mRNA expression was found to be associated with tumor diameter and lymph node metastasis. Gene enrichment analysis suggests that CBX3 is involved in the cell cycle and P53 signaling pathways. Aberrant expression of CBX3 in LUAD is correlated with DNA copy number alteration. In summary, our data imply that CBX3 plays an important role in the promotion of LUAD and may thus have potential as a prognostic biomarker and molecular therapeutic target for the disease.

## Graphical abstract

We report here that CBX3 (Chromobox 3) may be an independent prognostic biomarker in lung adenocarcinoma (LUAD). High expression levels of CBX3 are implicated in the cell cycle and p53 pathway, as well as tumor proliferation in LUAD. Copy number alterations are correlated with aberrant CBX3 expression in LUAD .

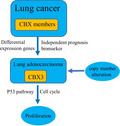

**Abbreviations**
CBX: Chromobox
CCLE: Cancer Cell Line Encyclopedia
CNA: copy number alteration
DFI: disease‐free interval
DSS: disease‐specific survival
GEO: Gene Expression Omnibus
GSEA: gene enrichment analysis
GTEx: The Genotype‐Tissue Expression
KEGG: Kyoto Encyclopedia of Genes and Genomes
LUAD: lung adenocarcinoma
LUSC: lung squamous cell carcinoma
NSCLC: non‐small‐cell lung cancer
NTL: normal lung tissue
OS: overall survival
Pc: polycomb
PFI: progression‐free interval
RFS: recurrence‐free survival
SD: standard deviation
TCGA: The Cancer Genome Atlas
TF: transcription factor
TIIC: tumor‐infiltrating immune cell

Lung cancer is a type of malignant tumor that induces considerable mortality and morbidity worldwide [1]. More than 85% of lung cancers are non‐small‐cell lung cancer (NSCLC), of which lung squamous cell carcinoma (LUSC) and lung adenocarcinoma (LUAD) are the two major categories [2]. Surgical resection, chemotherapy and radiation therapy are common therapies for early‐stage NSCLC, but all of these treatments are followed with undesirable side effects [3]. Lately, personalized precision therapy, which explicitly targets carcinoma cells according to the genetic alterations of patients and has apparently limited adverse effects compared with common therapies, had recently become a research hotspot [4]. However, given all of these treatments, most patients with NSCLC still have a poor 5‐year survival rate of 15.9% [2]. Hence exploiting biomarkers with greater potential for prognosis of LUAD is an emergency.

Chromobox (CBX) family members are typical components of the polycomb (Pc) inhibitory complex 1, an epigenetic regulatory complex that modifies chromatin to suppress transcription of target genes [5]. Until now, there were eight CBX family proteins described in the human genome that had been based on their single N‐terminal chromodomain [6]. All CBX family members are subdivided into two kinds: (a) heterochromatin protein 1 contained CBX1, CBX3 and CBX5 with an N‐terminal chromodomain; and (b) Pc contained CBX2, CBX4, CBX6, CBX7 and CBX8 with a conserved N‐terminal chromodomain and a C‐terminal Pc repressor box [6]. Also, different CBX family members bind to distinct regions of the chromatin, leading to specific transcription of target genes [5]. Increasing evidence had demonstrated that CBX family members play a key role in the initiation, progression and development of tumors via repressing differentiation and promoting self‐renewal of cancer stem cells [5, 7, 8]. CBX family members have been reported as aberrantly expressed in various tumors and have significant prognostic value. In breast cancer, overexpressed CBX1, CBX2 and CBX3 mRNA expression was correlated with worse recurrence‐free survival (RFS) [9]. In hepatocellular carcinoma, abnormal mRNA expressions of CBX1–3, CBX6 and CBX8 were independent prognostic predictors for shorter overall survival (OS), and the mutation rate of CBX family members was up to 51%, which was also observed to be negatively associated with OS and DFS [10]. Several studies were performed about the function of CBX members in lung cancer. CBX3 was highly expressed in NSCLC and associated with poor prognosis. Also, CBX3 promoted the proliferation of LUAD via regulating cell cycle and inhibiting apoptosis [11, 12, 13]. CBX7 was significantly down‐regulated in human lung carcinomas [14]. These studies mainly focused on studying individual genes in the CBX family. Global investigation about the contribution of specific CBX family members in tumorigenesis of NSCLC still remained to be clarified.

In the current research, we performed a comprehensive analysis for the expression and prognostic values of CBX family members in NSCLC and identified CBX3, which is the only member to function as an independent prognostic biomarker for LUAD. Also, CBX3 was predicted to regulate the proliferation and lymph node metastasis of LUAD.

## Materials and methods

### TCGA‐GTEx lung cancer cohort

The Cancer Genome Atlas–The Genotype‐Tissue Expression (TCGA‐GTEx) Lung Cancer data were downloaded from the University of California Santa Cruz Xena Browser (https://xenabrowser.net). Deleting the recurrence cancer samples, a total of 1359 lung cancer and normal cancer samples [513 primary LUAD and 59 solid tissue normal, 498 primary LUSC samples and 51 solid tissue normal, and 289 normal lung tissue (NTL)] remained, which were used for analysis of the mRNA expression of CBX family members and clinical parameter analysis. Among them, 500 primary LUAD cases contained OS and progression‐free interval (PFI) data, 465 contained disease‐specific survival (DSS) data, and 299 contained disease‐free interval (DFI) data; 494 primary LUSC cases with OS data, 441 with DSS data and 299 with DFI data were selected for further prognosis analysis. A total of 513 LUAD samples with intact clinical parameters were used to analyze the correlation between CBX3 mRNA expression and clinical parameters. The copy number alteration (CNA) of TCGA patients with LUAD and lung cancer cell lines in Cancer Cell Line Encyclopedia (CCLE) and methylation data of TCGA patients with LUAD were also downloaded from the University of California Santa Cruz Xena Browser.

### Survival analysis in Gene Expression Omnibus

GSE31210 of the Gene Expression Omnibus (GEO) database (https://www.ncbi.nlm.nih.gov/geo/) was used to validate the prognostic value of CBX3.

### Genomes enrichment analysis of CBX3

Genes coexpression with CBX3 was extracted using Pearson's correlation analysis (|*r*| ≥ 0.3) in cBioPortal (http://www.cbioportal.org) with the TCGA LUAD cohort. Kyoto Encyclopedia of Genes and Genomes (KEGG) pathways analysis was conducted in the DAVID website (https://david.ncifcrf.gov/summary.jsp). Gene sets were considered significantly enriched at predefined *P* values. Gene enrichment analysis (GSEA; https://www.gsea‐msigdb.org/gsea/index.jsp) was used to further identify the pathways that correlate to gene expression. A normalized enrichment score was calculated as the primary GSEA statistic. Gene sets were considered significantly enriched at predefined *P* values and false discovery rate < 0.25.

### Tumor‐infiltrating immune cells and immune genes correlated with the CBX3 via the tumor immune estimation resource database

Correlations between all tumor‐infiltrating immune cells (TIICs) and CBX3 were studied by the Tumor Immune Estimation Resource platform (https://cistrome.shinyapps.io/timer/), which owns web tools for gene‐specific correlational analysis with TIICs. TIICs included B cells, CD4^+^ T cells, CD8^+^ T cells, dendritic cells, macrophages and neutrophils, and tumor purity was used for the correction of Spearman based on correlation analysis. Immune gene was first extracted from Immpor (https://www.immport.org), and the correlation between CBX3 mRNA expression and immune genes in LUAD was then conducted.

### Prediction of target genes and miRNAs for CBX3

Potential target genes and up‐regulation miRNAs of CBX3 were predicted in starBase (http://starbase.sysu.edu.cn/rnaRNA.php?source); the transcription factors (TFs) that regulated CBX3 mRNA expression were predicted via PROMO (http://alggen.lsi.upc.es/cgi‐bin/promo_v3/promo/promoinit.cgi?dirDB=TF_8.3). All of the results were visualized using NetworkAnalyst (http://www.networkanalyst.ca). The network miRNAs were selected with interaction n > 5, and target genes with free energy < 40.

### Statistical analysis

graphpad prism version 8.0 (Graphpad, CA, USA) or r 3.6.3 version [Bell Laboratories (formerly AT&T, now Lucent Technologies) by John Chambers and colleagues] was used for data analysis. If not specifically mentioned, values in this study are presented in the form of mean ± standard deviation (SD). Two‐group independent sample comparisons Student's *t*‐test (two‐tailed) was used when the two groups had equal SDs, while Student's *t*‐test with Welch's correlation was used for unequal SDs. Multigroup samples statistics used one‐way ANOVA if the variances were equal; if not, Welch's ANOVA was performed. Bonferroni *post hoc* tests were carried out for all ANOVAs. Samples from TCGA LUAD cohort were divided into two groups, according to median values. OS, PFI, DSS and DFI curves were plotted using the Kaplan–Meier method, and OS, PFI, DSS and DFI differences were assessed using the log rank test. Pearson’s correlation analysis was used to evaluate the correlations among CBX3 mRNA expression and CNA. *P* < 0.05 was considered to indicate a statistically significant difference: ***P < 0.0001, **P < 0.01, *P < 0.05.

## Results

### CBX family members are significantly elevated in LUAD and LUSC tissues compared with NTLs

**Fig. 1:**
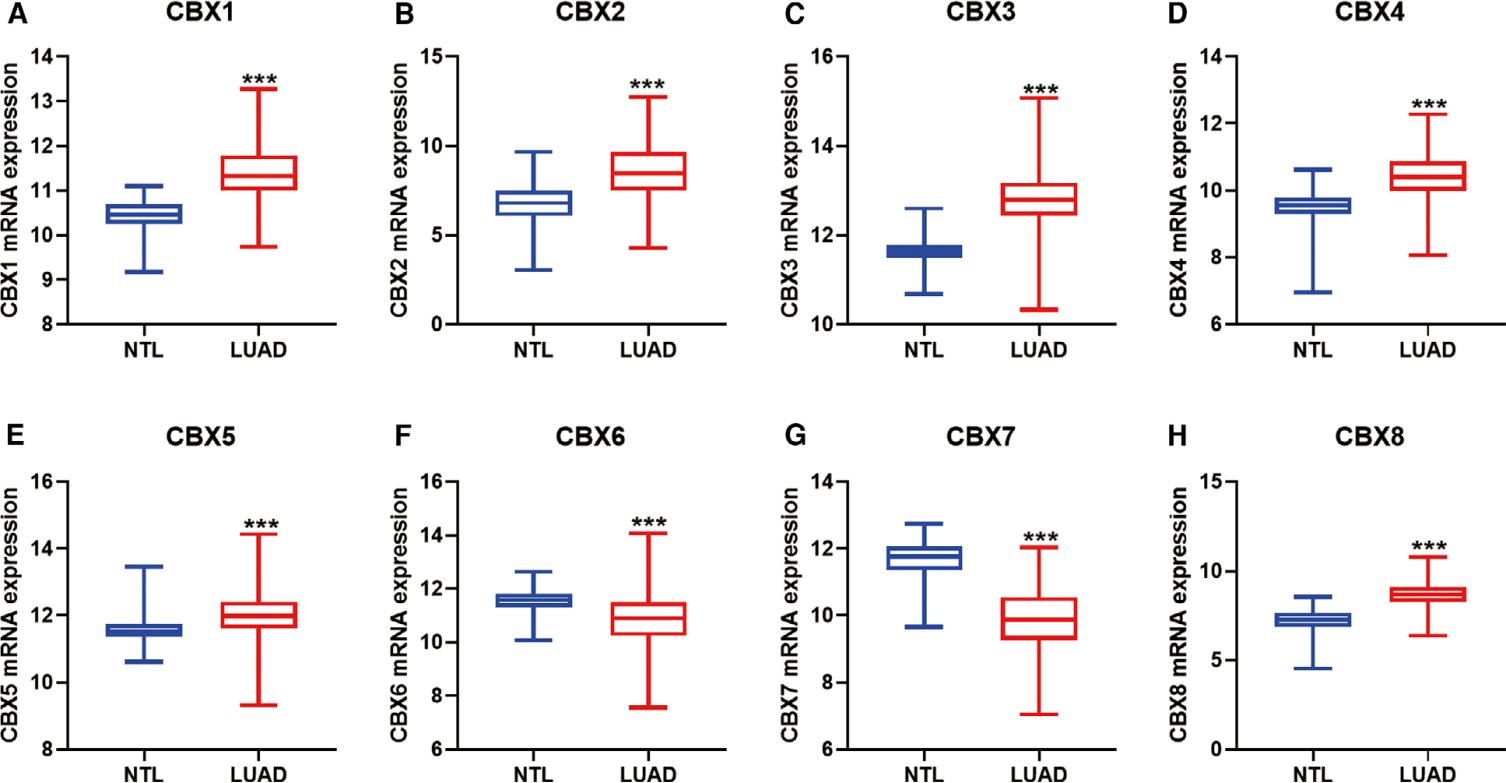
mRNA expression of CBX family members in TCGA‐GTEx LUAD tissues. (A–E, H) CBX1–5 and CBX8 mRNA was significantly increased in LUAD compared with NTL. (F, G) CBX6 and CBX7 mRNA was notably decreased in LUAD compared with NTLs. *P* < 0.05 was considered to indicate a statistically significant difference: ***P < 0.0001.

**Fig. 2:**
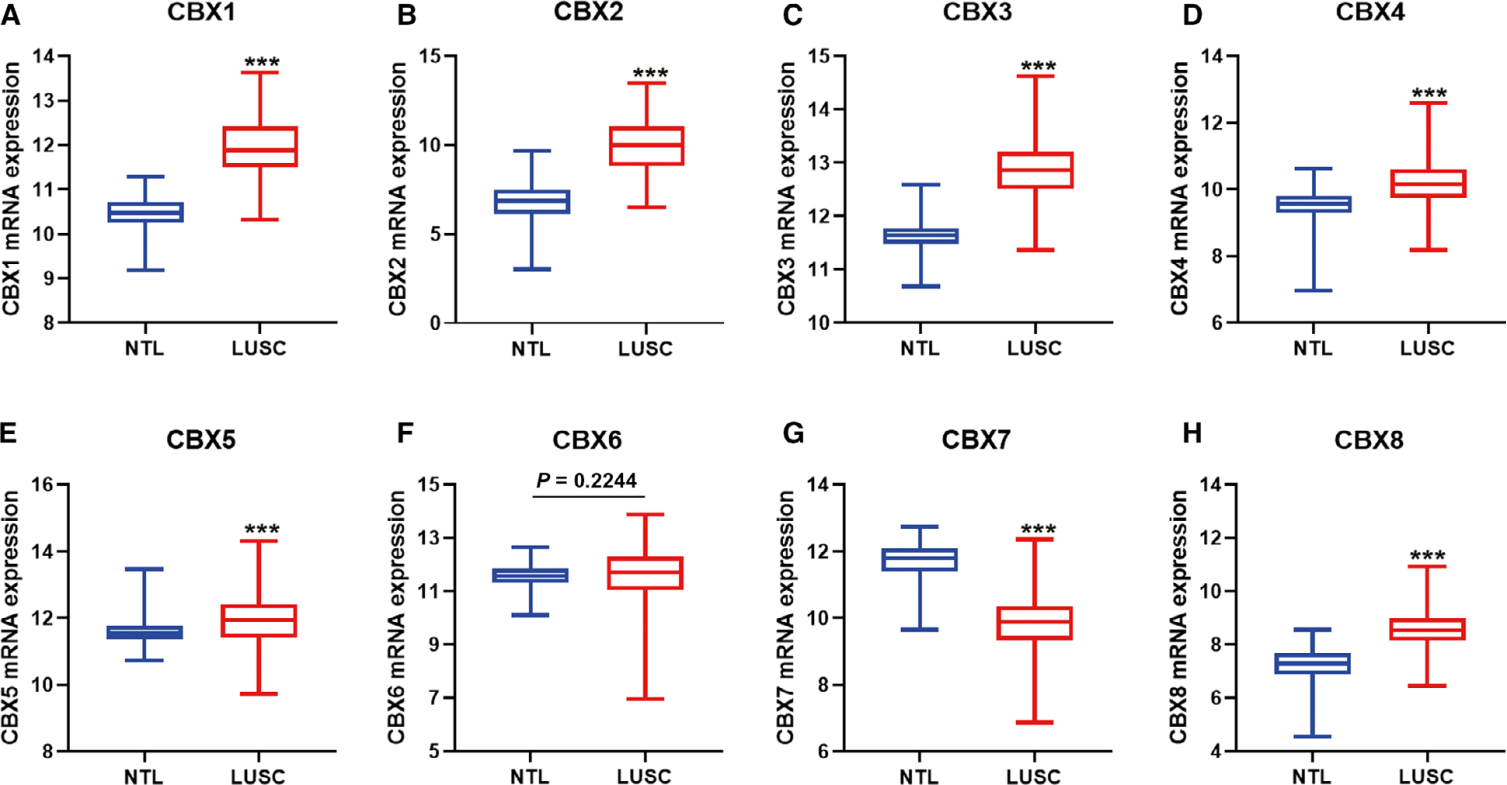
mRNA expression of CBX family members in TCGA‐GTEx LUSC tissues. (A–E, H) CBX1–5 and CBX8 mRNA was significantly increased in LUSC tissues compared with NTLs. (F). CBX6 showed no difference in LUSC and NTL. (G) CBX7 was notably decreased in LUSC tissues compared with NTLs. *P* < 0.05 was considered to indicate a statistically significant difference: ***P < 0.0001.

Expression levels of CBX family members were individually explored in the TCGA‐GTEx lung cancer cohort. The results showed that CBX1–5 and CBX8 were significantly elevated, whereas CBX6 and CBX7 were decreased in LUAD compared with NTL (Fig. 1). In LUSC, CBX1–5 overexpressed, whereas CBX7 and CBX8 decreased compared with NTL (Fig. 2). Surprisingly, CBX6 showed no difference between LUSC and NTL.

### CBX3 of CBX family members serve as an independent prognostic factor in LUAD

**Fig. 3:**
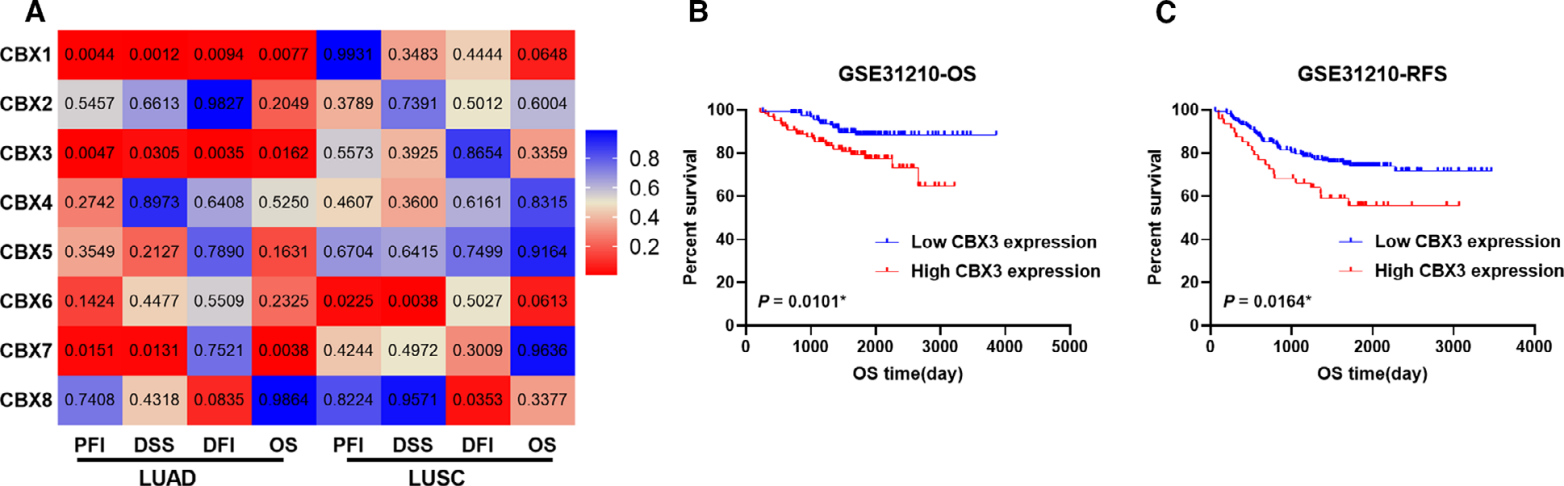
Association between CBX family members and prognostic value in TCGA patients with LUAD/LUSC. (A). Heatmap presented the log rank test results of PFI, DSS, DFI, and OS Kaplan–Meier analysis in TCGA patients with LUAD/LUSC. (B, C) Validation of the prognosis value of CBX3 in GSE31210. *P* < 0.05 was considered to indicate a statistically significant difference: *P < 0.05.

To identify potential promising biomarkers in the CBX family members, we explored the correlations between the CBX family members and OS, DSS, DFI and PFI in the TCGA LUAD and LUSC cohort. The results demonstrated that elevated CBX1 and CBX3 in patients with LUAD were negatively associated with OS, DSS, DFI and PFI (Fig. 3A). CBX2, CBX4–6 and CBX8 indicated no association with OS, DSS, DFI and PFI, whereas CBX7 correlated with OS, DSS and PFI, but not DFI. In LUSC, no CBX family members were concurrently correlated with OS, DSS, DFI and PFI (Fig. 3A). The Cox regression, both univariate and multivariate processes, was performed to validate whether CBX1 and CBX3 can serve as an independent prognosis and progression biomarker in LUAD. From the results (Table 1), we verified that CBX3, but not CBX1, can be a potential predictor for LUAD OS and PFI, rather than DSS and DFI.

**Table 1:** The univariate and multivariate analyses of OS, PFI, DFI and DSS of CBX1 and CBX3 and clinicopathological features in TCGA patients with LUAD. HR, hazard ratio.

Clinical parameters	OS: univariate Cox		OS: multivariate Cox		PFI: univariate Cox		PFI: multivariate Cox	
	HR	*P* Value	HR	*P* Value	HR	*P* Value	HR	*P* Value
Age	0.998 (0.980–1.017)	0.855			0.993 (0.976–1.040)	0.423	0.996 (0.979–1.014)	0.669
Sex	0.642 (0.767–1.56)	0.064			0.985 (0.701–1.375)	0.931		
Smoking	1.049 (0.895–1.229)	0.555			1.183 (1.006–1.390)	0.042*	1.217 (1.033–1.433)	0.019*
T	1.659 (1.341–2.051)	< 0.0001***	1.325 (1.028–1.709)	0.030*	1.398 (1.133–1.726)	0.245	1.183 (0.874–1.601)	0.277
N	2.387 (1.684–3.384)	< 0.0001***	1.591 (0.951–2.661)	0.077	1.501 (1.063–2.119)	0.021*	0.996 (0.586–1.693)	0.988
M	1.906 (1.071–3.390)	0.028*	0.874 (0.361–2.114)	0.765	1.444 (0.777–2.119)	0.002**	0.721 (0.278–1.865)	0.499
Stage	1.564 (1.331–1.838)	< 0.0001***	1.317 (0.914–1.898)	0.139	1.300 (1.102–1.534)	0.0002**	1.285 (0.885–1.866)	0.187
CBX1	1.211 (0.891–1.646)	0.220	1.158 (0.845–1.586)	0.362	1.245 (0.929–1.668)	0.143	1.183 (0.874–1.601)	0.277
CBX3	1.445 (1.060–1.970)	0.020*	1.431 (1.022–2.003)	0.037*	1.584 (1.176–2.134)	0.002**	1.567 (1.138–2.157)	0.006*

Clinical parameters	DFI: univariate Cox		DFI: multivariate Cox		DSS: univariate Cox		DSS: multivariate Cox	
	HR	*P* Value	HR	*P* Value	HR	*P* Value	HR	*P* Value
Age	1.017 (0.989–1.046)	0.239	1.014 (0.986–1.043)	0.314	0.978 (0.956–1.001)	0.064	0.991 (0.968–1.015)	0.461
Sex	1.029 (0.607–1.744)	0.916			0.911 (0.573–1.450)	0.695		
Smoking	1.136 (0.894–1.443)	0.298	1.192 (0.932–1.524)	0.162	1.142 (0.923–1.414)	0.220	1.200 (0.964–1.497)	0.103
T	1.442 (1.006–2.066)	0.046*	1.898 (1.121–3.216)	0.017*	1.600 (1.191–2.149)	0.002*	1.299 (0.915–1.844)	0.143
N	0.605 (0.313–1.172)	0.137	0.791 (0.271–2.311)	0.668	2.294 (1.444–3.645)	0.0004**	1.567 (0.777–3.160)	0.209
M					2.369 (1.176–4.771)	0.016*	1.355 (0.390–4.705)	0.632
Stage	0.811 (0.532–1.237)	0.330	0.701 (0.320–1.536)	0.375	1.564 (1.264–1.934)	< 0.0001***	1.222 (0.740–2.017)	0.434
CBX1	1.437 (0.927–2.227)	0.105	1.523 (0.986–2.352)	0.058	1.484 (0.996–2.211)	0.052	1.447 (0.950–2.204)	0.085
CBX3	1.459 (0.912–2.334)	0.115	1.370 (0.820–2.290)	0.229	1.512 (0.993–2.304)	0.054	1.487 (0.925–2.391)	0.102

* *P* < 0.05,

** *P* < 0.001,

*** *P* < 0.0001.

### Validation of prognosis value of CBX3 in GEO

GEO datasets GSE31210, which contained 226 LUAD samples with intact clinical information, were applied to validate the prognostic value of CBX3. As expected (Fig. 3B,C), CBX3 mRNA expression was negatively correlated with OS and RFS in LUAD. Altogether, these findings confirm that CBX3 is a prospective prognostic biomarker for LUAD.

### Correlation of CBX3 mRNA expression with clinicopathological features in LUAD

**Fig. 4:**
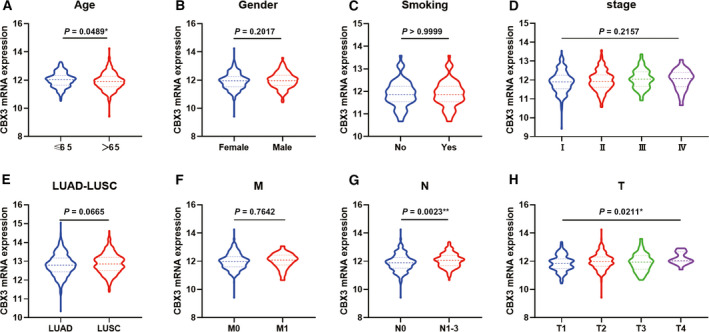
The correlation between CBX3 mRNA expression and clinical parameters in LUAD. (A, G, H). CBX3 mRNA expression was related with age, tumor diameter and lymph node metastasis of LUAD. (B–F). CBX3 mRNA expression showed no relation with sex, smoking history, histopathological classification, distant metastasis and TNM stage of LUAD (*n* = 512). *P* < 0.05 was considered to indicate a statistically significant difference: **P < 0.01, *P < 0.05.

To delve into the role of CBX3 expression in LUAD progression, we assessed the association of clinicopathological features with CBX3 mRNA expression (Fig. 4). Our study divulged that CBX3 mRNA expression was related with age, tumor diameter and lymph node metastasis, whereas no relation was found with sex, smoking history, histopathological classification, distant metastasis and TNM stage in the TCGA‐LUAD cohort. Hence it was hypothesized that CBX3 may promote proliferation of LUAD.

### KEGG pathway enrichment analysis of CBX3

**Fig. 5:**
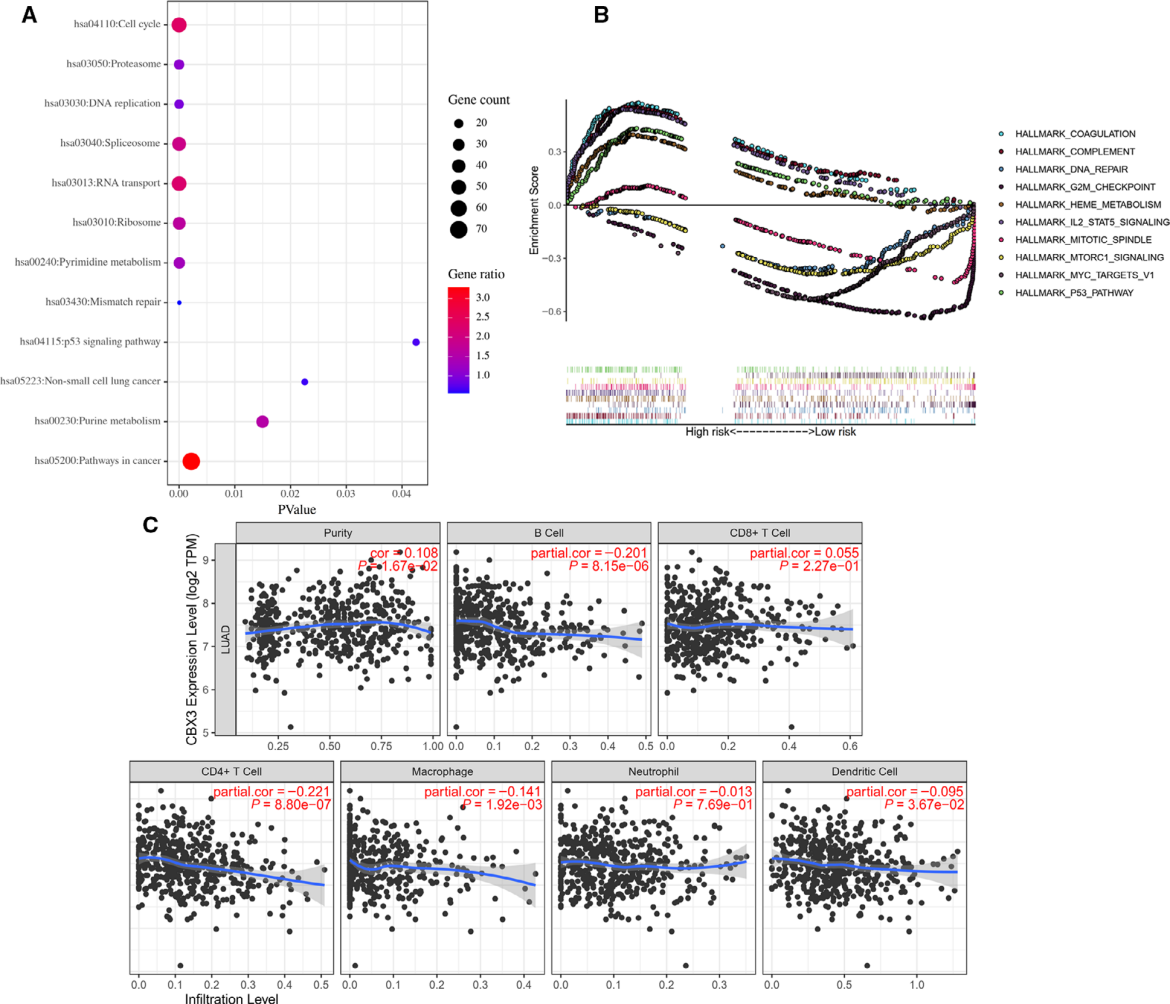
The function of CBX3 in LUAD. (A) GSEA‐based KEGG pathways used coexpression genes. (B) GSEA analysis reveals potential pathways that CBX3 implicated. (C) The relation between CBX3 mRNA expression and each type of TIIC (B cells, CD4^+^ T cells, CD8^+^ T cells, neutrophils, macrophages and dendritic cells). *P* < 0.05 was considered to indicate a statistically significant difference.

To further test the function and pathways of CBX3 involved in LUAD, we performed the KEGG enrichment analysis by DAVID. First, we analyzed the coexpression genes of CBX3 in cBioPortal (Table S1). Genes with |*r*| ≥ 0.3 were further used to perform KEGG pathways analysis in DAVID (Table S2). Figure 5A lists representative pathways in which CBX3 participates. GSEA, which contained another algorithm based on MSigDB, was further used to validate the pathways that CBX3 involved (Table S3). The results indicated that CBX3 was involved in cell‐cycle‐related G2M checkpoint, the mitotic spindle biological process, interleukin‐2–STAT3 pathways, etc. (Fig. 5B). Compared with the results of the two enrichment analyses, we found that both of them indicated CBX3 was implicated in regulating cell cycle, P53 signaling pathways.

In view of the growing correlation between immunological features and cancer prognosis, we then examined the association between TIICs and CBX3 in LUAD. In fact, CBX3 found the highest correlation between CD4^+^ T cells (Fig. 5C). Moreover, we analyzed the correlation between CBX3 mRNA expression and immune genes (Table S4). PPIA, a peptidyl‐prolyl cis‐trans isomerase A, showed the greatest correlation with CBX3.

### Prediction of target genes and the up‐regulation factor for CBX3

**Fig. 6:**
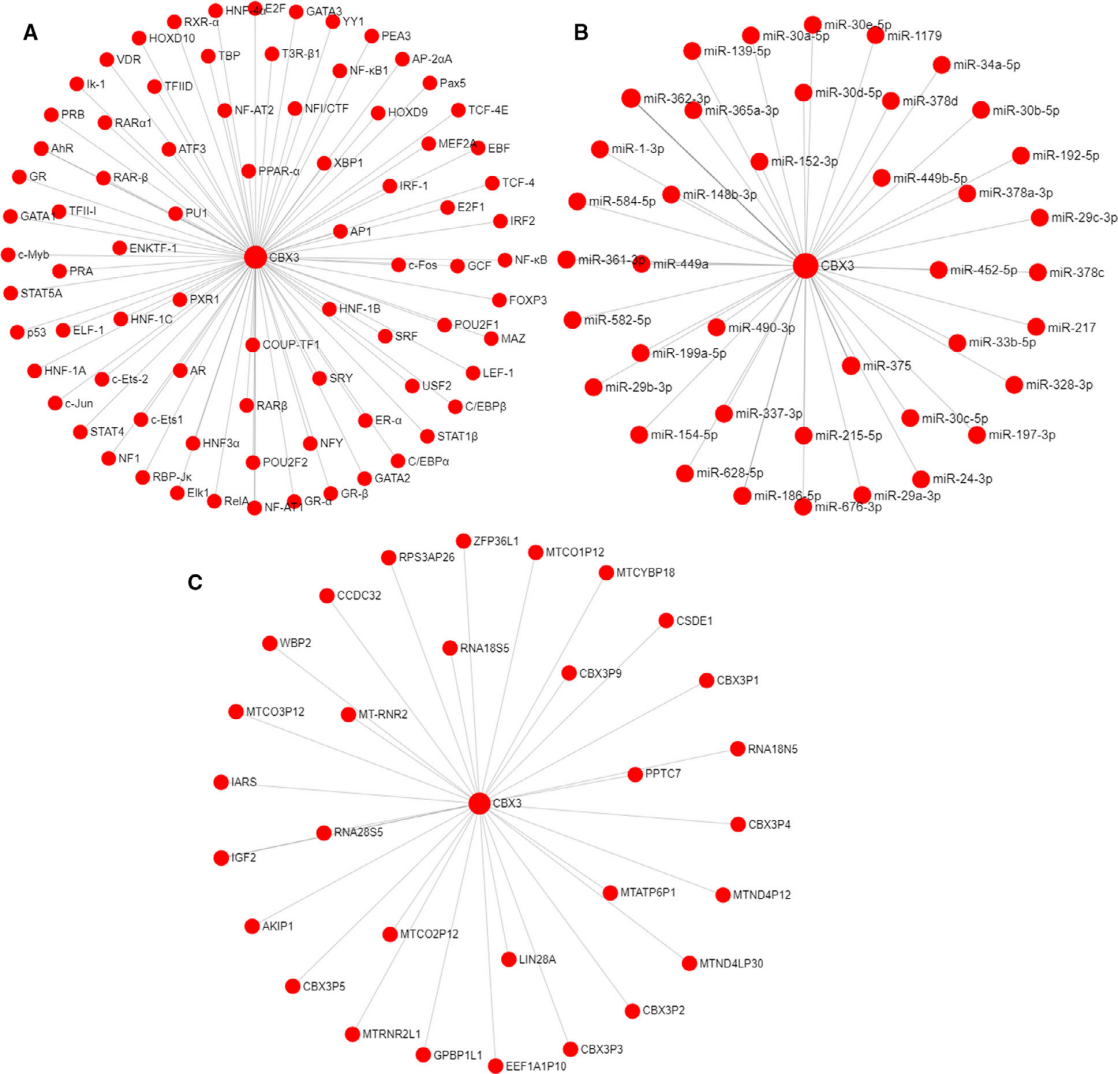
TFs, miRNA and target genes prediction of CBX3. (A) The predicted networks of TFs and CBX3 in PROMO. (B) The predicted networks of miRNAs and CBX3 in starBase. (C) The predicted networks of target genes of CBX3 in starBase.

To clarify the mechanism of aberrant expression of CBX3 in LUAD, we analyzed the regulation network of CBX3 from target genes, the upstream miRNAs and TFs in starBase. From the results, we found 49 miRNAs and 81 TFs were predicted to bind to the promoter of CBX3, which then regulated its transcription (Fig. 6A,B). Moreover, 320 genes, including protein, pseudogene and long noncoding RNA, may be regulated by CBX3, and the genes with free energy < 40 were placed in Figure 6C.

### DNA CNA is associated with CBX3 mRNA expression in LUAD

**Fig. 7:**
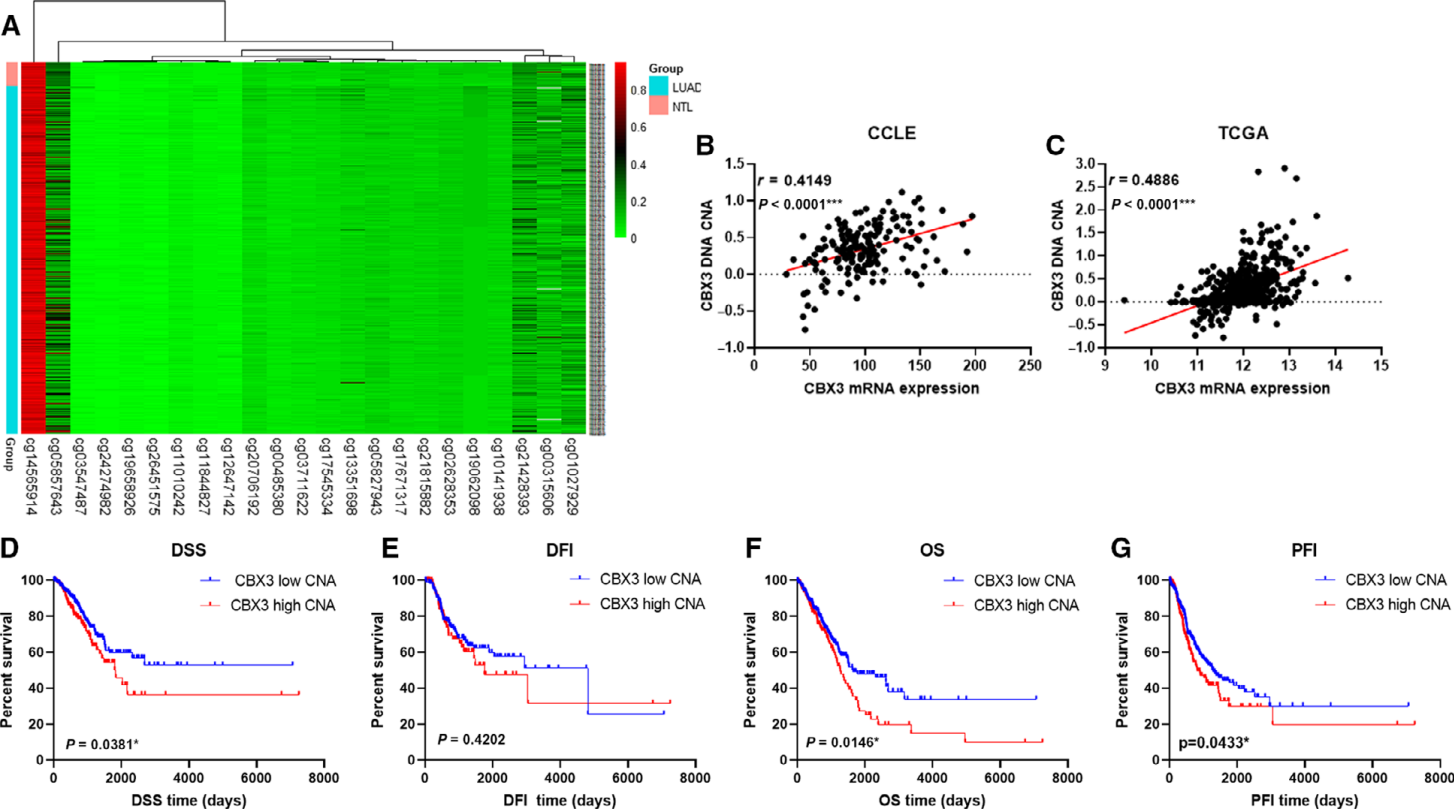
Correlation of CBX3 mRNA expression and its DNA methylation and CNA in TCGA patients with LUAD (*n* = 512) and CCLE lung cancer cell lines (*n* = 172). (A) DNA methylation of CBX3 in LUAD and NTL. (B, C) Correlation analysis between CBX3 mRNA expressions is its CNA both in LUAD tissue and lung cancer cell lines. (D–G) Kaplan–Meier analysis between CBX3 CNA and DSS, DFI, OS and PFI. *P* < 0.05 was considered to indicate a statistically significant difference: ***P < 0.0001, *P < 0.05.

Epigenetic regulation is another accepted way of regulating gene expression. Consequently, we analyzed the CNA and methylation of CBX3. Methylation of CBX3 is shown in Fig. 7A; all of the methylation sites in LUAD were not significantly altered compared with normal tissues. Besides, analysis between CNA and CBX3 mRNA suggested they strongly positively correlated (Fig. 7B,C). Surprisingly, Kaplan–Meier analysis demonstrated high CBX3 CNA was negatively associated with DSS and OS, and positively associated with PFI, whereas no relation with DFI was found (Fig. 7D–G).

## Discussion

Besides aberrant cancer genetics, epigenetic regulation, which occurred in all stages of lung cancer, proved to be implicated in lung cancer occurrence and progression. Studies have shown that stage‐specific epigenetic changes can serve as powerful and reliable tools for early diagnosis and prognosis monitoring, as well as effective therapeutic targets for patients with lung cancer [15]. CBX family members, which are essential ingredients of epigenetic regulation, are involved in the progression of various tumors, including NSCLC [15, 16]. In previous studies, several CBX family members have been proved to play important roles in NSCLC. Alam *et al*. [12] demonstrated that CBX2–4 were highly expressed in LUAD, and overexpression of CBX3 is associated with poor prognosis. Zhou *et al*. [13], Han *et al*. [17] and Chang *et al*. [11] also found CBX3 to be overexpressed in NSCLC, and Zhou *et al*. [13] further confirmed that CBX3 is an independent prognostic biomarker for NSCLC. In the study by Hu *et al*. [18], CBX4 had been shown to be overexpressed in lung cancer compared with adjacent normal tissues. CBX5 is proved to be up‐regulated in LUAD compared with nontumorous lung tissues in the study of Yu *et al*. [19], and its overexpression is associated with shorter OS. However, CBX7 is remarkably decreased in human lung cancer cells [14]. Results in our study first demonstrated the eight CBX family members' expression in NSCLC. Consistent with the previous study, CBX3–5 showed increased expression and CBX7 decreased in LUAD and LUSC, respectively. Also, we found elevated CBX1, CBX2 and CBX8 in LUAD and LUSC, and decreased CBX6 in LUAD, but no difference in LUSC. Because there are few studies on the prognostic value of the CBX family in LUAD and LUSC, we have analyzed it thoroughly in TCGA datasets. Results of the prognosis analysis demonstrated that CBX3 was the only member to affect OS, DSS, DFI and PFI in LUAD, but not LUSC, which is somewhat contrary from the study of Zhou *et al*. [13]. Univariate and multivariate regression analyses identified CBX3 as an independent prognostic factor for OS and PFI in LUAD. Validation in GSE31210 further confirmed CBX3 was associated with OS and RFS of LUAD. According to these results, we concluded CBX3 is the best prognosis predictor among CBX family members for LUAD, and we will focus on CBX3 for further mechanism study.

Further investigations between CBX3 expression and clinical parameters demonstrated CBX3 may be implicated in the proliferation and lymph node metastasis. Also, gene enrichment pathway analysis found that CBX3 regulated cell cycle. These predictions are in accordance with the research of Alam *et al*. [12] and Zhou *et al*. [13], which certified that CBX3 regulated the proliferation of lung cancer by regulating the G1/S phase transition of cell cycle and inhibiting apoptosis. In addition, enrichment analysis also demonstrated that CBX3 may be involved in the regulation of PI3K‐Akt, Ras signaling pathways, and so on. At present, no research has been conducted about the pathways CBX3 involved in the lung cancer, and our prediction is worth further verification through *in vitro* experiments.

The study by Sun *et al*. [20] found Cbx3/HP1γ insufficiency mice were treated with Cbx3/HP1γ‐insufficient CD8^+^ T cells, which caused changes of tumor immune environment, alleviated the tumor burden and, in turn, suppressed the tumor growth. Hence we speculated that CBX3 may play a role in tumor immunity. The correlation between CBX3 and TIICs was explored, which signified that CBX3 was correlated with the infiltration of B cells and CD4^+^ T cells.

Although various studies had been conducted on the mechanism of CBX3 in tumor, the systematic analysis about the upstream regulators and target genes of CBX3 had not been reported yet. In this study, we investigated the upstream miRNA and TFs that regulate CBX3 mRNA expression and the target genes of CBX3. Also, several upstream and target genes of CBX3 had been proved in cancer [21, 22].

The mechanism of gene dysregulation in NSCLC is complex. Among all of the mechanisms, genetic and epigenetic alterations, including CNA, DNA methylation and somatic mutations, commonly cause aberrant gene expression accompanied by anomalous cancer cell behavior. Therefore, we analyzed epigenetic alteration of CBX3 in TCGA LUAD dataset, including DNA methylation and CNA. The results displayed that aberrant expression of CBX3 may be related with CNA, rather than DNA methylation. What is more surprising is that CNA of CBX3 is related with DSS, OS and PFI, which further prove that CBX3 is a good prognostic marker for LUAD.

## Conclusions

Our study is the first to clarify the potential effect of CBX family members, particularly CBX3. We found that CBX1 and CBX3 were simultaneously correlated with OS, DSS, DFI and PFI, which prove their significant prognostic value in LUAD. Moreover, our analysis demonstrated that CNA is associated with the mRNA expression of CBX3, and the DSS, OS and PFI of LUAD. In addition, GSEA analysis indicated that CBX3 may be involved in regulating the proliferation of LUAD. The constraint of our research was the absence of experimental verification and clinical samples' cohort validation. In the future, experimental studies regarding the molecular mechanism of CBX3 in lung cancer will be performed.

## Conflict of interest

The authors declare no conflict of interest.

## Author contributions

XX and XC conceived and designed the study. XX and YN performed the bioinformatics analysis and wrote the paper. JL and HW viewed and checked the manuscript.

## Supporting information


**Table S1**. Coexpression genes of CBX3 in TCGA LUAD from cBioPortal.

**Table S2**. GSEA results of CBX3 in TCGA LUAD‐based KEGG pathways.

**Table S3**. GSEA analysis results of CBX3 in TCGA LUAD.

**Table S4**. Correlation between CBX3 and immune genes mRNA expression in TCGA LUAD.

## Data Availability

The datasets used during this study are available from the corresponding author upon reasonable request. Data were obtained from TCGA (http://portal.gdc.cancer.gov), GEO database (https://www.ncbi.nlm.nih.gov/geo/) CCLE (https://portals.broadinstitute.org/ccle/about); cBioPortal (http://www.cbioportal.org) and the University of California Santa Cruz Xena Browser (https://xenabrowser.net).
